# Discordant pair analysis for sample efficient model evaluation

**DOI:** 10.1038/s41598-023-48017-4

**Published:** 2023-11-28

**Authors:** Donald Musgrove, Andrew Radtke, Tarek Haddad

**Affiliations:** Medtronic Inc., 8200 Coral Sea St NE, Mounds View, MN 55112 USA

**Keywords:** Machine learning, Cardiology

## Abstract

We present a new technique for assessing the effectiveness of a classification algorithm using discordant pair analysis. This method utilizes a known performance baseline algorithm and a large unlabeled dataset with an assumed class distribution to obtain overall performance estimates by only assessing the subset of examples that the algorithms classify discordantly. Our approach offers an efficient way to evaluate the performance of an algorithm that minimizes the human adjudications needed while also maintaining precision in the evaluation and in some cases improving the evaluation quality by reducing human adjudication errors. This approach is a computationally efficient alternative to the traditional exhaustive method of performance evaluation and has the potential to improve the accuracy of performance estimates. Simulation studies show that the discordant pair method reduces the number of adjudications by over 90%, while maintaining the same level of sensitivity and specificity.

## Introduction

Supervised machine learning models require large quantities of labeled data to optimize and validate performance. The quality of these data labels is especially important in applications such as medical diagnostics, where the model decisions can have large impacts on patient outcomes. Ensuring proper quality relies on sourcing data labels from certified professionals, which is both expensive and time consuming. Thus, in resource constrained environments, it becomes necessary to seek to minimize the number of data labels required to characterize a machine learning model’s performance. A separate but related issue arises when updating machine learning models, by training new data either on an existing model or on a completely new modeling architecture. In both cases, characterizing the new model’s performance requires a completely new set of data labels for the validation dataset. That is, in many regulatory settings, reusing validation datasets across model updates is not feasible due to the potential for data leakage and concerns around generalizability^1^. Another issue that arises with high performing models, challenging cases become critical for comparing model quality. These challenging cases can be difficult to find and may require large amounts of manual adjudications before enough edge cases are obtained and labeled to have an accurate quantification of performance of any model. A final issue is related to the regulatory burden associated with medical diagnostics. In many cases a full description and pre-specification of the validation data label acquisition approach is necessary, including potential sample sizes and desired effect sizes^2^. To address each of these issues, we are proposing a novel technique for achieving a reduced size validation dataset using a so-called discordant pair analysis based on the model predictions from a baseline and new, updated model—with a discordant pair analysis, we use the off-diagonals of the 2 × 2 confusion matrix, where the predictions of a baseline and updated model disagree.

Previous methods for improving or reducing the number of data labels have involved ranking schemes or active learning approaches that used the model being trained to subsample data requiring labels. In^3^, a label cleaning method is proposed that iteratively ranks data instances based on the estimated label correctness and labelling difficulty associated with each sample. Based on the ranking scheme, annotators relabel data until a budget is exhausted, where the budget can be time and/or monetary based. This active learning model can be applied to the use case of data labeling in the context of model validation, but the methodology was developed with the existence of noisy labels in mind, rather than no labels at all.^4^ proposed an active learning framework that is a sample efficient technique that the authors name active testing. With the active testing approach, sample points are selected for labeling based on maximizing the accuracy of an empirical risk estimate.

In both described approaches, the underlying methodology relies on labeling data based on the quality of existing labels or maximizing some performance metric. In a high regulatory burden setting, where pre-specification of all validation details is required, the label cleaning approach of^3^ would not reduce the number of labels requiring adjudication. Similarly, the active testing approach of^4^ is not applicable since all sample points would need to be selected for labeling to maximize accuracy—random subsampling is not a preferred approach in a high regulatory burden environment.

In this paper, we begin by precisely defining the discordant pair analysis technique. We apply our approach to the updating of a neural network model used for the classification of atrial fibrillation (AF) in electrocardiograms (ECG) collected from implantable cardiac monitors (ICMs)^5^. The goal in this use case is to replace an existing model running in a production environment with a better performing model that has statistically significantly superior specificity for detecting AF. Next, using several simulation studies, we analyze the impact of several assumptions on the number of expert adjudications required and the updated model performance. We finish the paper with a discussion of the approach, the potential limitations, and future work.

## Methods

**Figure 1:**
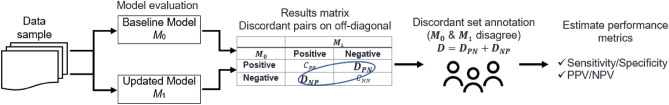
Overview of the discordant pair analysis.

In this work, we develop a method for reducing the number of model validation samples required for labeling based on disagreement between a baseline model and a new, updated model, where the baseline model has known performance that is expected to generalize to the validation data. Importantly, this method is developed for binary labels. An overview of the approach is shown in Fig. 1.

The discordant pair method proceeds as follows. We begin with an estimate of the prevalence of the binary outcome, $PREV$, a sample size, $n$, the estimated number of positive outcomes $P=n\times PREV$, and a performance requirement definition, along with statistical power considerations, of the desired sensitivity of the updated model; see, e.g.,^6^ for further sample size considerations around diagnostic metrics. Thus, we procure a total sample size of $n$ samples. The $n$ samples are evaluated similarly to an A/B testing framework, where each of the samples is evaluated on both the baseline and updated models. Next, results are collected into a 2 × 2 matrix, as shown in Table 1.

**Table 1:** Baseline model (${M}_{0}$) and updated model (${M}_{1}$) 2 × 2 results table.

Baseline model—${M}_{0}$	Updated model—${M}_{1}$	
	Outcome = positive	Outcome = negative
Outcome = positive	${C}_{PP}$	${D}_{PN}$
Outcome = negative	${D}_{NP}$	${C}_{NN}$

In this framework, we identify binary model outcomes from two models as concordant, $C={C}_{PP}+{C}_{NN}$, or discordant, $D={D}_{NP}+{D}_{PN}$, and, of course, the total sample size is $n=C+D$. We can further decompose each of the four paired concordance and discordant outcomes as

$${C}_{PP}=T{P}_{0C}+F{P}_{0C}+T{P}_{1C}+F{P}_{1C},$$

$${C}_{NN}=T{N}_{0C}+F{N}_{0C}+T{N}_{1C}+F{N}_{1C},$$

$${D}_{NP}=T{N}_{0D}+F{N}_{0D}+T{P}_{1D}+F{P}_{1D},$$

$${D}_{PN}=T{P}_{0D}+F{P}_{0D}+T{N}_{1D}+F{N}_{1D},$$

where $T{P}_{ij}$, $T{N}_{ij}$, $F{P}_{ij}$, and $F{N}_{ij}$ are the true positive, true negative, false positive, and false negative counts, respectively, $i\in \left(0, 1\right)$ indexes the baseline and updated models, respectively, and $j\in \left(C, D\right)$ indexes the concordant and discordant sets, respectively. As an illustrative example to help guide understanding, $T{P}_{0C}$ is the number of true positive outcomes from the baseline model in the concordant set.

Recall, at this stage none of the samples are labeled. We are proposing to label only the discordant outcomes where the models disagree, i.e., samples that contribute to ${D}_{NP}$ and ${D}_{PN}$, to facilitate a final estimate of the updated model performance. To see this, we can estimate the sensitivity of the updated model, $SEN{S}_{1}$, using the following approach. Begin by assuming a sensitivity for the baseline model, $SEN{S}_{0}$, estimated from a previous validation or performance surveillance effort. Thus, we assume:

$$SEN{S}_{0}=\frac{{TP}_{0}}{P}=\frac{{TP}_{0C}+ {TP}_{0D}}{P} ,$$

and solving for ${TP}_{0C}$ gives

$${TP}_{0C}=SEN{S}_{0}\times P- {TP}_{0D}.$$

We must have that ${TP}_{0C}={TP}_{1C}$, that is, the number of true positives between the two models in the concordant only set are equivalent. Then, we can estimate the sensitivity of the updated model as

$$\begin{array}{lll}SEN{S}_{1}& =& \frac{T{P}_{1}}{P},\\ & =& \frac{{TP}_{1C}+{TP}_{1D}}{P},\\ & =& \frac{\left(SEN{S}_{0}\times P-T{P}_{0D}\right)+T{P}_{1D}}{P},\end{array}$$

where $P$ is the estimated number of binary outcomes and $T{P}_{i}$, $i\in (\mathrm{0,1})$, is the number of true positives from model $i$. This derivation shows that the sensitivity of the updated model can be calculated using only the baseline model sensitivity, positive outcome prevalence, and values from the discordant set.

We can use a similar application to estimate the specificity of the updated model, $SPE{C}_{1}$, as well. Assuming a specificity for the baseline model, ${SPEC}_{0}$, we have:

$${SPEC}_{0}=\frac{{TN}_{0}}{N}=\frac{{TN}_{0C}+ {TN}_{0D}}{N} ,$$

where $N=n-P$ is the assumed number of negative binary outcomes. Solving for ${TN}_{0C}$ gives

$${TN}_{0C}={SPEC}_{0}\times N- {TN}_{0D},$$

where, ${TN}_{0C}$ is the number of true negative outcomes from the baseline model in the concordant set. Similar to the sensitivity calculation, we have that ${TN}_{0C}={TN}_{1C}$, i.e., the number of true negatives between the two models in the concordant set are equivalent. Then, we can estimate the specificity of the updated model, $SPE{C}_{1}$, as

$$\begin{array}{lll}{SPEC}_{1}& =& \frac{T{N}_{1}}{N},\\ & =& \frac{{TN}_{1C}+{TN}_{1D}}{N},\\ & =& \frac{\left({SPEC}_{0}\times N-T{N}_{0D}\right)+T{N}_{1D}}{N}.\end{array}$$

We’ve thus demonstrated that the specificity of the updated model can be calculated using only the baseline model specificity, negative outcome prevalence, and values from the discordant set.

### Confidence intervals

Beyond point estimates of the sensitivity and specificity, we may be required to estimate confidence intervals, as is often the case in a regulatory setting where non-inferiority or superiority of an updated model is to be demonstrated^7^. The sensitivity and specificity metrics depend in part on assumptions based on data sets collected in the past, and so a bootstrapping approach^8^ can be used to propagate uncertainties and obtain confidence intervals for the sensitivity and specificity of the updated model.

The bootstrapping approach uses multi-stage Monte Carlo sampling and proceeds as follows. Beginning with baseline model sensitivity and specificity values, $SEN{S}_{0}$ and $SPE{C}_{0}$, respectively, and positive outcome prevalence $PREV$, for the $k$ th Monte Carlo sample, $k=1,\dots ,K$, we have:

$$\begin{array}{lll}PRE{V}_{k}& \sim & Beta\left(100, 100/PREV-100\right),\\ {P}_{k}& \sim & Binom\left(n,PRE{V}_{k} \right),\\ T{P}_{0k}& \sim & Binom\left({P}_{k}, SEN{S}_{0}\right),\\ SEN{S}_{1k}& \sim & Beta\left(T{P}_{0k}-T{P}_{0D}+T{P}_{1D}+1,{P}_{k}-\left(T{P}_{0k}-T{P}_{0D}+T{P}_{1D}\right)+1\right),\end{array}$$

resulting in $K$ Monte Carlo samples of the updated model sensitivity. We then sample the updated model specificity:

$$\begin{array}{lll}{N}_{k}& =& n-{P}_{k}\\ T{N}_{0k}& \sim & Binom\left({N}_{k}, SPE{C}_{0}\right),\\ SPE{C}_{1k}& \sim & Beta\left(T{N}_{0k}-T{N}_{0D}+T{N}_{1D}+1,{N}_{k}-\left(T{N}_{0k}-T{N}_{0D}+T{N}_{1D}\right)+1\right),\end{array}$$

where, in general, $Beta\left(A,B\right)$ is a beta distribution with rates $A$ and $B$, and $Binom\left(C,D\right)$ is a Binomial distribution with $C$ trials and probability $D$. Upon sampling each $SEN{S}_{1k}$ and $SPE{C}_{1k}$ we can compute upper and lower quantiles of the samples to estimate the confidence bounds, e.g., the 2.5th and 97.5th quantiles correspond to two-sided 95% confidence bounds. In this paper, we use $K=\mathrm{10,000}$ Monte Carlo samples.

## Results

### Real world example

The discordant pair analysis was applied to the validation of a cloud-based deep neural network model used as a tool for secondary screening of electrocardiogram (ECG) recordings^5^. Briefly, insertable cardiac monitors (ICMs) are implanted in patients requiring long-term cardiac monitoring. Upon detection of atrial fibrillation (AF) episodes in the heart rhythm, the ICM will transmit 2-min ECG recordings to a cloud-system for further screening by a deep neural network. The purpose of the neural network is to reduce the false positive AF detections that are ultimately shown to clinicians for review.

The baseline model was characterized during an initial validation effort^5^. During the initial characterization, the ICM’s AF episode-level prevalence was found to be 61.5%. The baseline model achieved a sensitivity of 98.8% and a specificity of 72.7% for detecting AF.

We next worked to improve the specificity of the baseline model by updating the model weights on a new dataset. Upon training an updated model, a completely independent dataset of 4,302 ICM-detected AF episodes from 771 patients was created over a 12-month period ending June, 2021. The baseline and updated models were evaluated on the 4,302 episodes. Table 2 shows the evaluation results. We can see that the discordant set has size $D={D}_{NP}+{D}_{PN}=307$.

**Table 2:** Evaluation results of baseline (${M}_{0}$) and updated (${M}_{1}$) models.

Baseline model—${M}_{0}$	Updated model—${M}_{1}$	
	Outcome = positive	Outcome = negative
Outcome = positive	${C}_{PP}=2640$	${D}_{PN}=272$
Outcome = negative	${D}_{NP}=35$	${C}_{NN}=1355$

Following our discordant pair approach, we began with a baseline model sensitivity and specificity of $SEN{S}_{0}=0.988$ and $SPE{C}_{0}=0.727$, respectively, and assumed positive and negative outcome prevalences of 2,645 and 1,657, respectively. Results are shown in Table 3. We estimate the updated model sensitivity as 99.1% (95% CI: 98.5%, 99.6%), and the updated model specificity as 87.5% (95% CI: 83.9%, 92.0%).

**Table 3:** Performance results for episode-level detection of AF.

	Sensitivity (95% CI)	Specificity (95% CI)	N adjudications
Discordant pair			
Updated model	99.1 (98.5, 99.6)	87.5 (83.9, 92.0)	307
Validation			
Baseline model	99.4 (98.6, 99.8)	81.4 (78.2, 84.3)	1372
Updated model	99.1 (98.2, 99.7)	88.9 (86.2, 91.2)	1372

To validate our approach, we created a 2^nd^ independent dataset of 1,372 ICM-detected AF episodes from 331 different patients than those collected for the discordant pair analysis. In this new data set all samples were adjudicated similarly to a traditional validation approach where each of the 1,372 episodes were labeled. Results are shown in Table 3. Our updated model achieved a sensitivity of 99.1% (95% CI: 98.2%, 99.7%) and a specificity of 88.9% (95% CI: 86.2%, 91.2%). Conversely, the baseline model achieved a sensitivity of 99.4% (95% CI: 98.6%, 99.8%) and a specificity of 81.4% (95% CI: 78.2%, 84.3%).

### Simulation study

To assess the efficacy of our approach, we carried out three simulation studies to investigate the (1) effect of correlation between the baseline and updated model predictions; (2) effect of sample size; and (3) effect of misspecification of the positive outcome prevalence. For each simulation study, we assumed sensitivity values of 0.988 and 0.990 for the baseline and updated models, respectively, and specificity values of 0.727 and 0.882 for the baseline and updated models, respectively. Correlated binary outcomes were simulated using a bivariate Gaussian copula with binomial marginals, see Supplementary Appendix for details.

### Effect of model correlation

**Figure 2:**
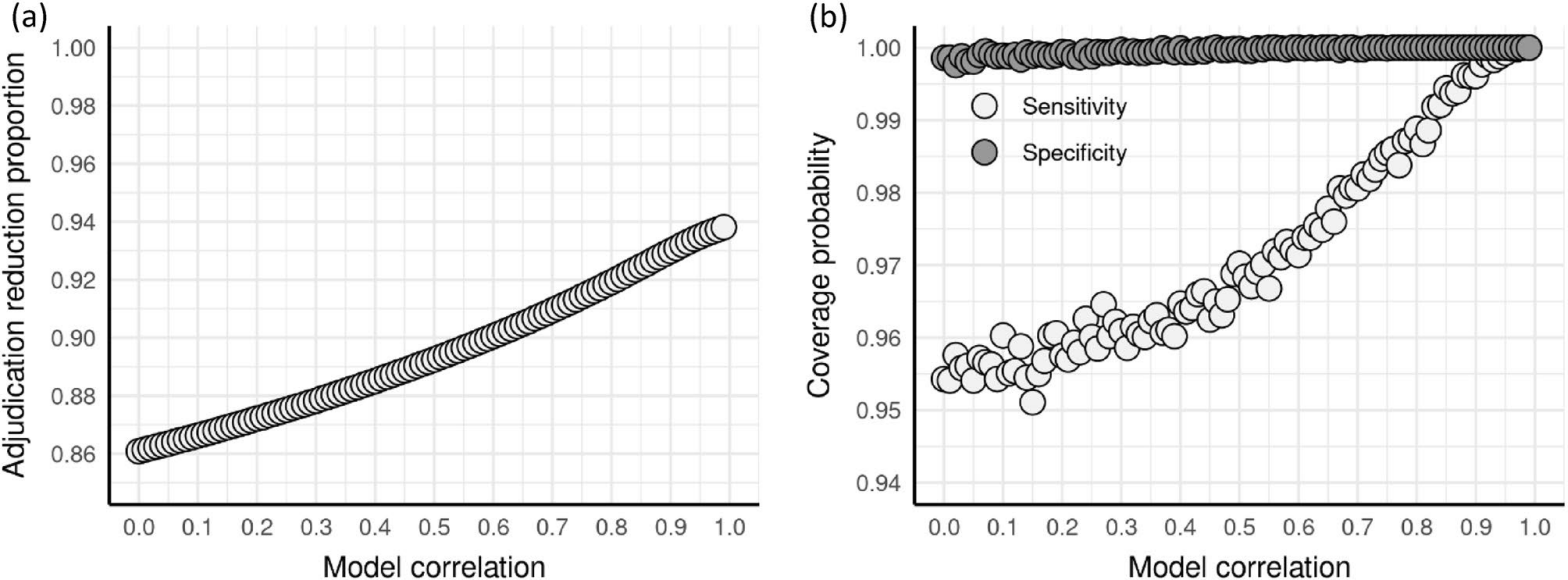
Effect of between-model correlation on (**a**) percent adjudication reduction sensitivity and (**b**) specificity confidence interval coverage probability.

To assess the effect of correlation between the baseline and updated model outcomes. We simulated 10,000 trials over correlation values ranging from 0 to 0.99. Similar to the previous simulation study, we assumed a positive outcome prevalence of 0.615, and now a constant sample size of 5000. As shown in Fig. 2*,* we found that the correlation primarily affects the percent reduction in number of adjudications needed and CI coverage probability. We see that the specificity CI coverage probability is at or above the nominal 95% coverage for all correlation values. The percent adjudication reduction is above 80% for all correlation values, with high correlation values achieving the greatest percent reduction in the number of adjudications needed at well over 90%.

### Effect of sample size

**Figure 3:**
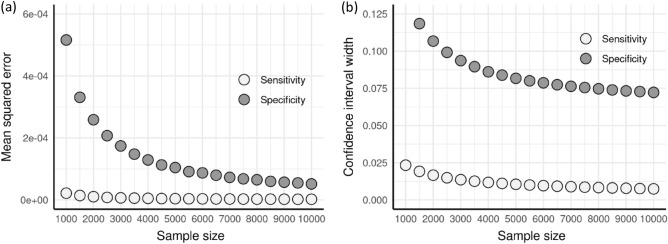
Effect of sample size on sensitivity and specificity (**a**) mean squared errors and (**b**) confidence interval widths.

To assess the effect of sample size on metric precision, we simulated 10,000 trials over samples sizes ranging from 1000 to 10,000 bivariate binary outcomes. For each trial, we assumed a positive outcome prevalence of 0.615 and a between model correlation of 0.90. As shown in Fig. 3, the sample size primarily affects the mean squared error (MSE) and confidence interval (CI) width of the sensitivity and specificity. With MSE, we compared the estimated metrics using the discordant pair analysis to the observed metric values. As expected, as the sample size increases, both MSE and CI width decreases. As the sample size approaches 5000, the MSEs drops below 0.0001 and the CI widths are below 0.08. We also investigated the effect of sample size on the CI coverage probability and percent reduction in number of adjudications needed but found no association. Complete results are shown in the Supplementary Appendix.

### Effect of misspecification of prevalence

**Figure 4:**
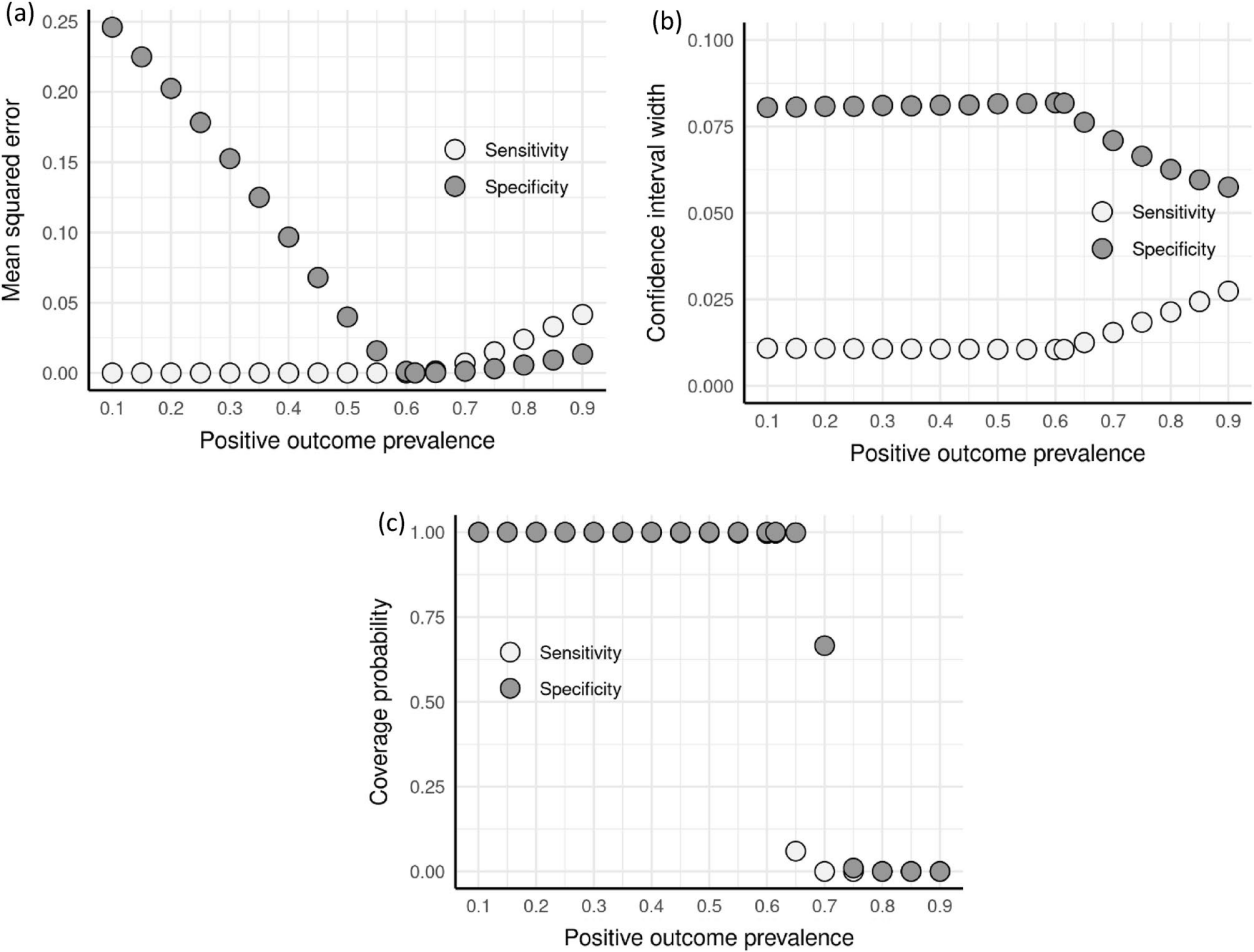
Effect of misspecification of positive outcome prevalence on sensitivity and specificity (**a**) mean squared error, (**b**) confidence interval width, and (**c**) coverage probability of the confidence intervals.

Finally, we assessed the effect of misspecification of the positive outcome prevalence. We simulated 10,000 trials over actual prevalence values ranging from 0.1 to 0.9, while the assumed prevalence was held constant at 0.615. Recall, this implies that we are assuming that the positive outcome prevalence is 61.5%, while the simulations allow for actual prevalence values ranging from 10 to 90%. For each trial, we assumed a between model correlation of 0.90 and a constant sample size of 5000. Figure 4 shows the effect of misspecified prevalence on each of MSE, CI width, and CI coverage probability.

Beginning with MSE, the sensitivity is relatively constant until the assumed prevalence of 0.615 is achieved, then the MSE of sensitivity increases, a pattern we also see repeated with the CI width. Meanwhile, the MSE of specificity decreases for prevalence values less than 0.615, until the assumed prevalence of 0.615 is achieved and remains relatively low as the prevalence increases. In contrast, the specificity CI width is relatively flat until the assumed prevalence is achieved, and then decreases as the prevalence increases. Finally, the coverage probabilities of both the sensitivity and specificity are high for low prevalence values but decrease sharply as the prevalence increases beyond the assumed prevalence of 0.615.

## Conclusion and discussion

Our application of the discordant pair analysis to a real-world example demonstrates the method’s ability to estimate important diagnostic metrics with a reduced adjudication sample size. In our analysis, we adjudicated only 307 of the 4302 total episodes, a 93% reduction in the number of required adjudications. An important metric that we estimated was the specificity, which in the discordant pair analysis was estimated at 87.5% (95% CI: 83.9%, 92.0%). In our separate validation analysis, we estimated the specificity at 88.9% (95% CI: 86.2%, 91.2%); the point estimates are very close, with the primary difference observed in the confidence interval limits and the discordant pair analysis results in wider confidence limits owing to the uncertainty characterization in many of the model inputs. We also saw a similar performance between the two estimates for the sensitivity—the sensitivity estimate from the discordant pair analysis was 99.1% (95% CI: 98.5%, 99.6%) and the sensitivity from the separate validation analysis was 99.1% (95% CI: 98.2%, 99.7%). With the comparison between the baseline and updated models, we are primarily interested in demonstrating superiority of the updated model specificity, compared to the baseline model. In the discordant pair analysis, the specificity lower confidence limit of the updated model, 83.9%, is much higher than the baseline model’s specificity of 72.7%, thus demonstrating superiority.

Turning to the simulation study results, for our assumed prevalence of 0.615, a total sample size near 5,000 was optimal for MSE and CI width. The effect of between-model correlation is beneficial here, we expect a relatively high model correlation, and so the coverage probability is at or near nominal. An additional benefit of high between-model correlation is the reduction in the number of adjudications needed: we can achieve greater than 90% reduction, a significant time and cost savings. When specifying the positive-outcome prevalence, a key takeaway is that optimal results are achieved when the value is over specified, but within 10 percentage points of the true underlying prevalence. Otherwise, the results are sensitive to prevalence values that are under specified. An important and related concept to misspecification of prevalence is the misspecification of the baseline model sensitivity and/or specificity, which we did not consider directly. This is due in part to the fact that the estimate of the updated model sensitivity or specificity is a function of both prevalence and baseline model sensitivity or specificity, respectively.

There are several limitations associated with this modeling approach. First, the baseline model assumptions, including sensitivity and specificity, rely on the temporal applicability of the baseline model. That is, data drift and, relatedly, adjudication drift, can be problematic sources that affect the usefulness of the baseline model, along with any assumptions drawn^9^. Second, the binary outcome prevalence assumption faces a similar challenge. The underlying patient population in which the ICMs are prescribed can potentially change over time, changing the prevalence of the binary outcome of interest. Last, the distributional assumptions for bootstrapping the confidence intervals may require closer examination depending on the application. A key driver of variability is the uncertainty around the prevalence of positive outcomes. With a prevalence of 0.615, we assumed a beta distribution with rate parameters 100, and 62.6, resulting in 99% of the values falling between 0.54 and 0.710. This spread of potential prevalence values is very realistic for our application but may need to be loosened or tightened for other applications.

A secondary use case for this approach is during active learning, where the most difficult cases can be identified and examined more closely^10^. Beyond active learning, there is also a practical perspective, where it is important to only label data the minimum amount of data necessary to accomplish the task at hand. Extended periods of performing repetitive tasks, including manual adjudications, can result in fatigue and a decrease in the quality of labels^11^. To ensure the highest quality of labeling, adjudicators should focus on the most challenging cases for shorter durations. However, it is difficult to determine the challenging cases a priori, though it is possible for such cases to arise after many hours of adjudication, increasing the likelihood of errors for these edge cases. This is a very important concept, because in many situations, the difficult edge cases have the highest value in distinguishing between two high-performing models. Therefore, the discordant pair analysis can be used as part of a strategy to maximize adjudicators’ time by focusing efforts on the challenging cases, resulting in reduced time and effort needed to evaluate a model and produce better performance results.

Finally, an important extension of this approach is to the case where there are multiple cases per patient. We ignored multiple cases per patient purely for illustrative purposes, but it is important to properly adjust estimates, especially around the construction of confidence intervals. Thus, future work will adapt generalized estimating equations^12^ or generalized linear mixed effects models^13^ to constructing confidence intervals.

### Supplementary Information

Supplementary Information.

## Data Availability

The heart rhythm data were obtained from an internal Medtronic data warehouse, so due to the sensitive nature of the research supporting data is not available publicly—but will be made available from corresponding author on reasonable request.
