# Supplementary material for: Discordant pair analysis for sample efficient model evaluation
Source: Sci Rep. 2023 Nov 28;13:20987. doi: 10.1038/s41598-023-48017-4 (PMC10684559; doi:10.1038/s41598-023-48017-4)
Supplement: Supplementary file 1 — Supplementary Information. [file 41598_2023_48017_MOESM1_ESM.docx]

# Appendix

## Simulating correlated binary outcomes

Correlated bivariate binary outcomes were simulated from a Gaussian copula (Ross, 2022) using the following steps:

1. Construct a 2x2 correlation matrix, i.e., $R=I\times\left( 1-\rho\right)-J\times\rho$, where $I$ is the $2x2$ identify matrix, $J$ is the $2x2$ matrix of ones, and $\rho$ is the bivariate correlation
2. Compute the Cholesky decomposition $L=chol\left( R \right)$, such that $L^{T} L=R$
3. Sample correlated normal values as $Y=ZL$, where $Z$ is a $n\times2$ matrix of standard normal samples
4. Let $X_{ij}=F_{j}^{-1}\left( \Phi\left( Y_{i} \right), p_{j} \right)$, $i=1,\ldots n$, $j=1,2$ be the correlated binary samples, where $\Phi$ is the cumulative distribution function (CDF) of a standard normal distribution, and $F_{j}^{-1}$ is the quantile function, or inverse CDF, of the binomial distribution, with 1 trial and a probability of $p_{j}$.

## Complete simulation study results

| (a) 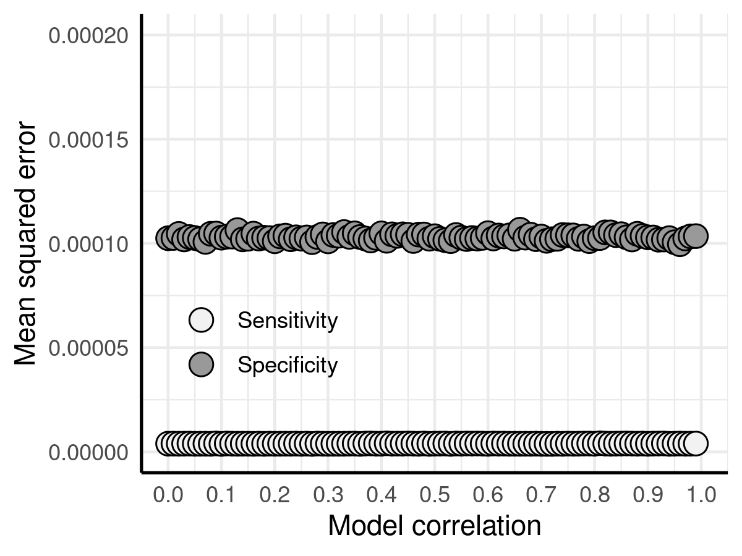 | (b) 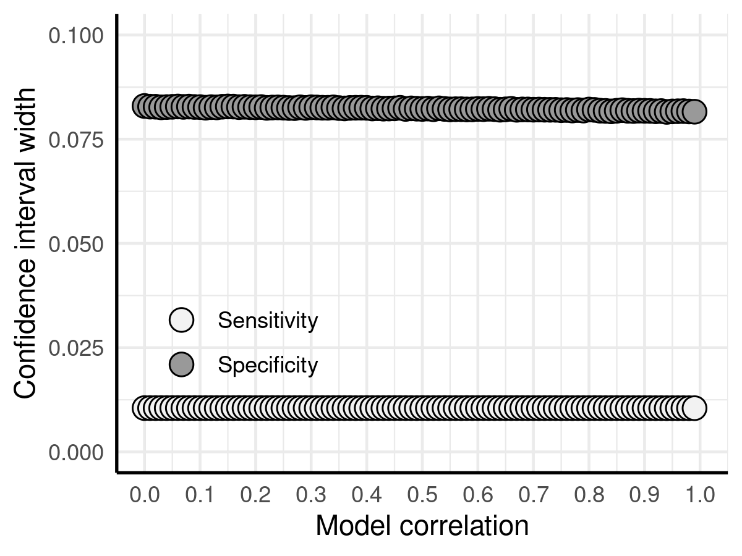 |
| --- | --- |
| (c) 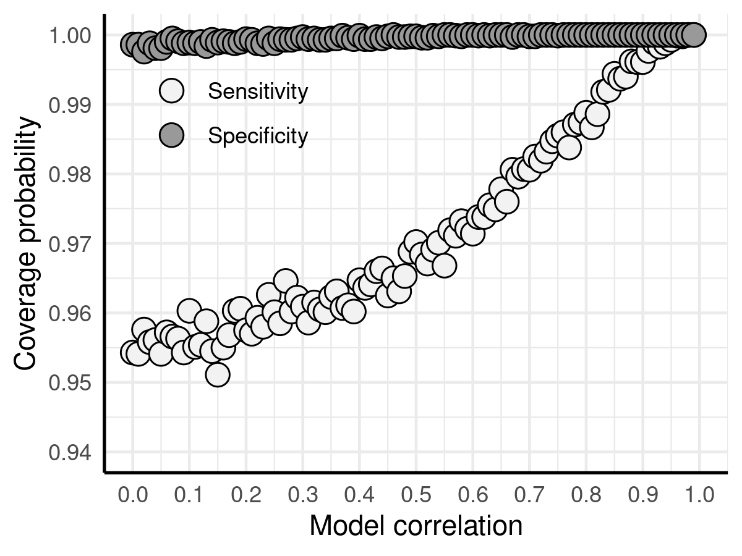 | (d) 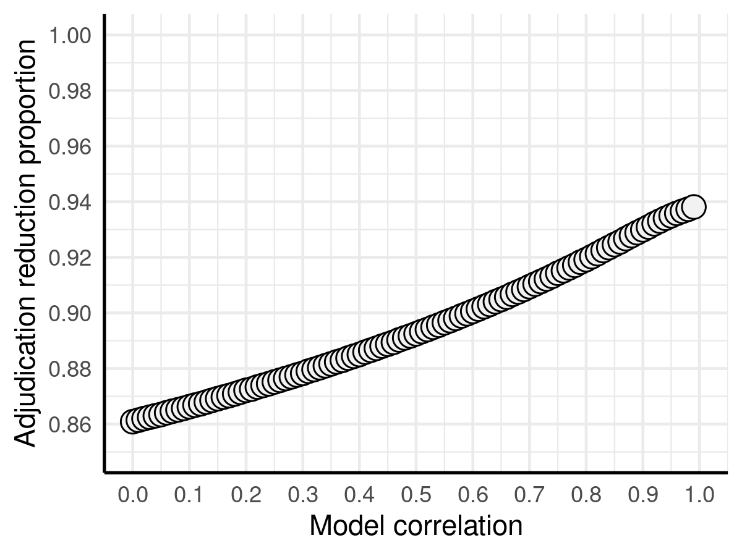 |

Figure S1. Effect of between-model correlation on sensitivity and specificity (a) mean squared errors; (b) confidence interval widths; (c) coverage probability of the confidence intervals; and (d) percent adjudication reduction.

| (a)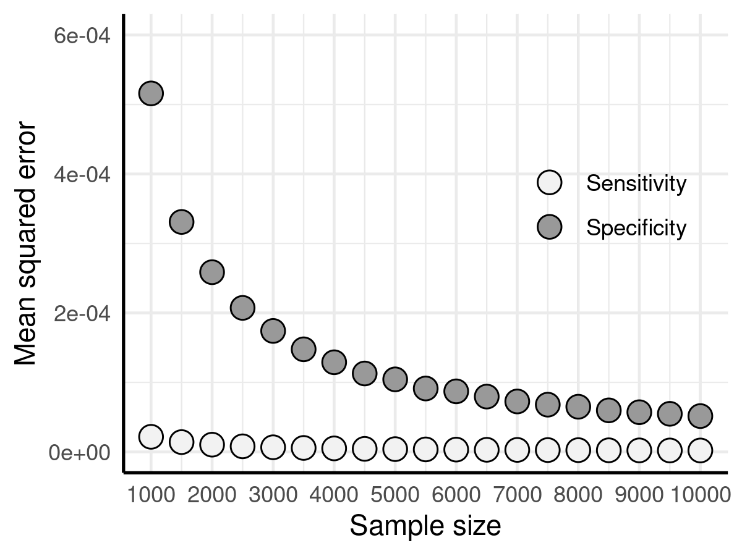 | (b)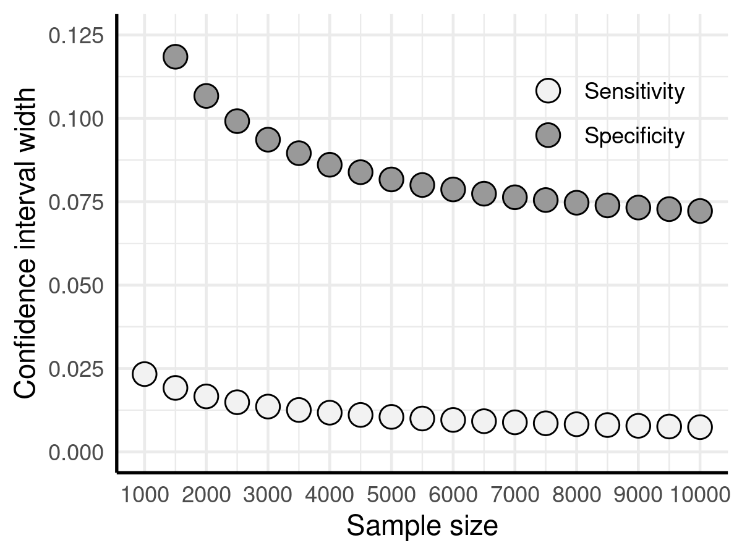 |
| --- | --- |
| (c) 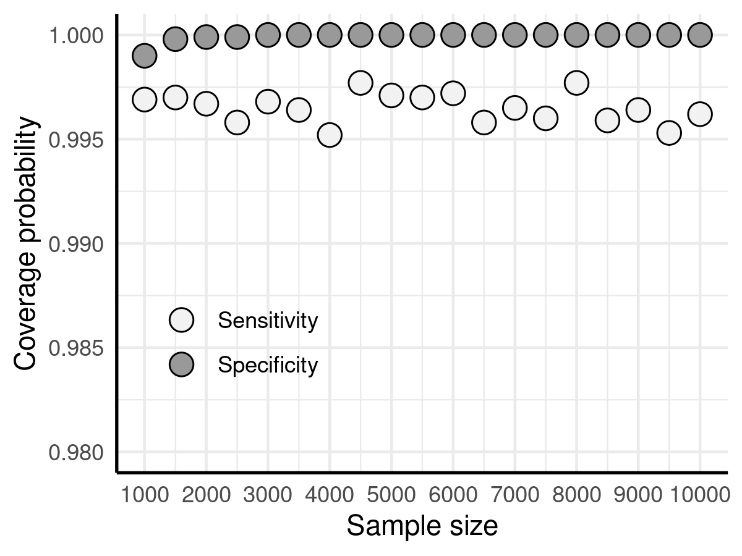 | (d) 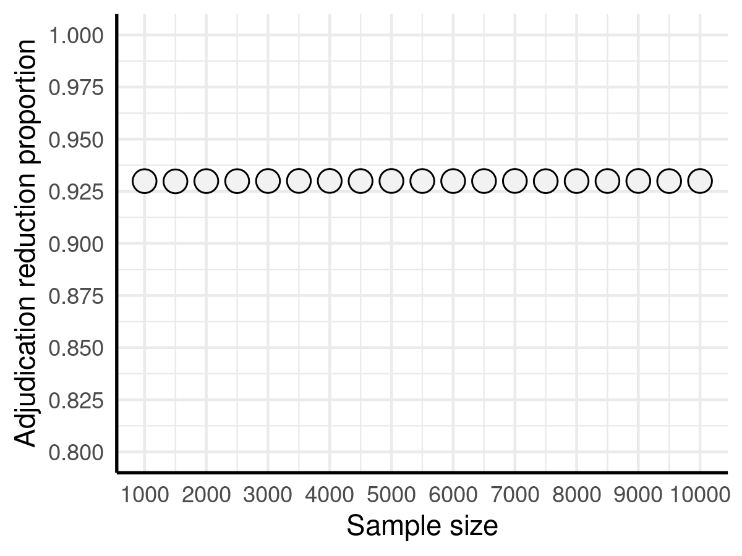 |

Figure S2. Effect of sample size on sensitivity and specificity (a) mean squared errors; (b) confidence interval widths; (c) coverage probability of the confidence intervals; and (d) percent adjudication reduction.

| (a) 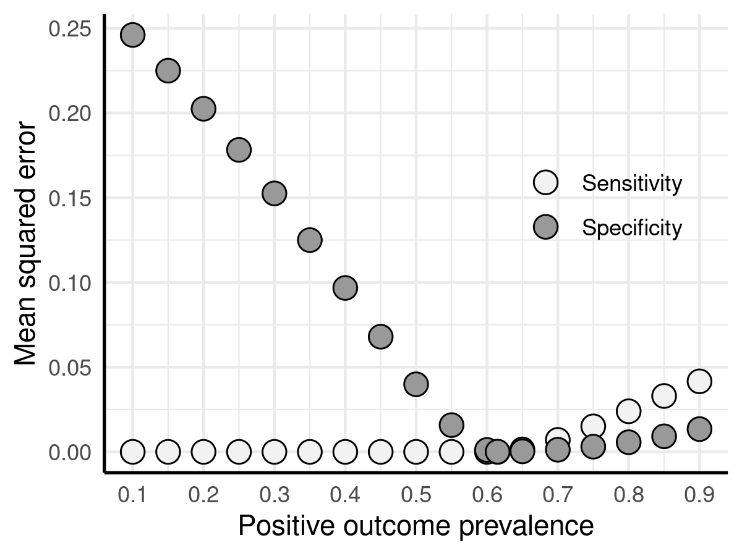 | (b) 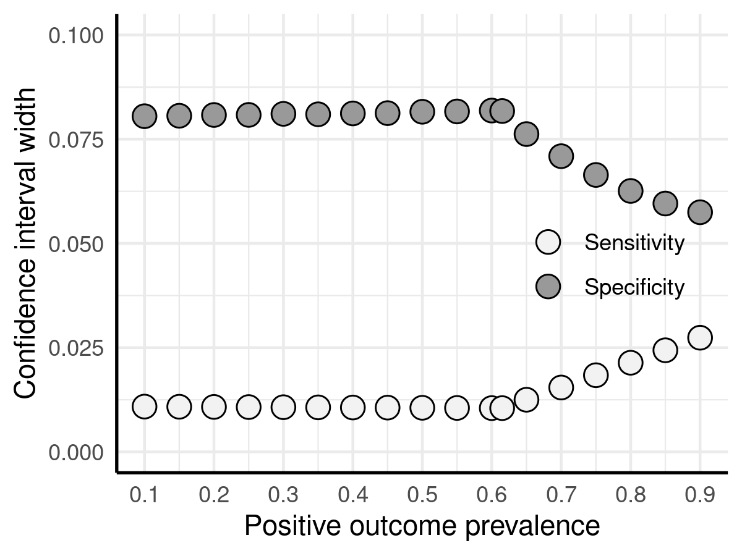 |
| --- | --- |
| (c) 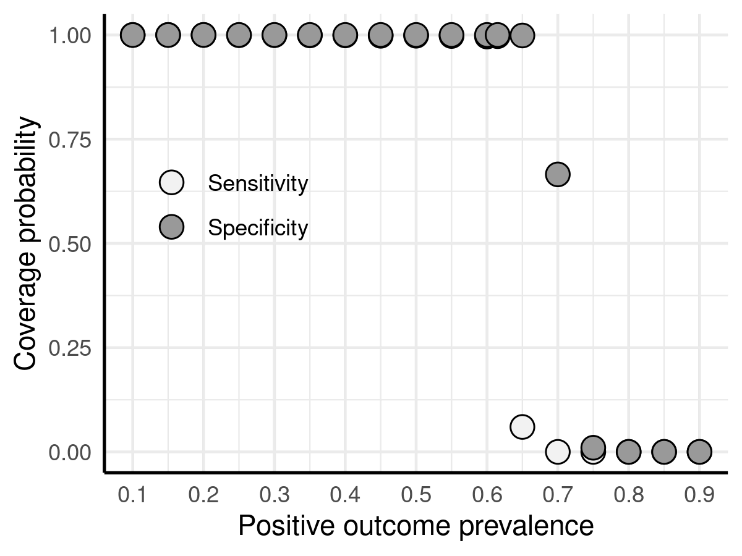 | (d) 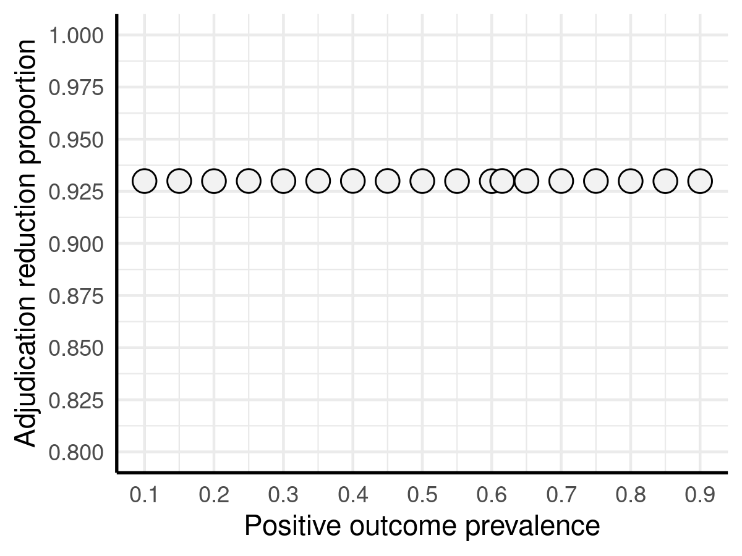 |

Figure S3. Effect of misspecification of positive outcome prevalence on sensitivity and specificity (a) mean squared errors; (b) confidence interval widths; (c) coverage probability of the confidence intervals; and (d) percent adjudication reduction.
